# The Splicing of the Mitochondrial Calcium Uniporter Genuine Activator MICU1 Is Driven by RBFOX2 Splicing Factor during Myogenic Differentiation

**DOI:** 10.3390/ijms23052517

**Published:** 2022-02-24

**Authors:** Denis Vecellio Reane, Cristina Cerqua, Sabrina Sacconi, Leonardo Salviati, Eva Trevisson, Anna Raffaello

**Affiliations:** 1 Department of Biomedical Sciences, University of Padova, 35131 Padova, Italy; denis.vecellioreane@unipd.it; 2 Clinical Genetics Unit, Department of Women’s and Children’s Health, University of Padova, Via Giustiniani 3, 35128 Padova, Italy; c.cerqua@irpcds.org (C.C.); leonardo.salviati@unipd.it (L.S.); 3 Istituto di Ricerca Pediatrica—IRP, Fondazione Città della Speranza, 35127 Padova, Italy; 4 Centre Hospitalier Universitaire de Nice, Peripheral Nervous System and Muscle Department, Rare Neuromuscular Disease Reference Center, ERN-Euro-NMD, Université Côte d’Azur (UCA), 06103 Nice, France; sacconi@unice.fr; 5 Myology Center, University of Padova, 35131 Padova, Italy

**Keywords:** mitochondrial calcium homeostasis, alternative splicing, skeletal muscle, myogenic differentiation

## Abstract

Alternative splicing, the process by which exons within a pre-mRNA transcript are differentially joined or skipped, is crucial in skeletal muscle since it is required both during myogenesis and in post-natal life to reprogram the transcripts of contractile proteins, metabolic enzymes, and transcription factors in functionally distinct muscle fiber types. The importance of such events is underlined by the numerosity of pathological conditions caused by alternative splicing aberrations. Importantly, many skeletal muscle Ca^2+^ homeostasis genes are also regulated by alternative splicing mechanisms, among which is the Mitochondrial Ca^2+^ Uniporter (MCU) genuine activator MICU1 which regulates MCU opening upon cell stimulation. We have previously shown that murine skeletal muscle MICU1 is subjected to alternative splicing, thereby generating a splice variant—which was named MICU1.1—that confers unique properties to the mitochondrial Ca^2+^ uptake and ensuring sufficient ATP production for muscle contraction. Here we extended the analysis of MICU1 alternative splicing to human tissues, finding two additional splicing variants that were characterized by their ability to regulate mitochondrial Ca^2+^ uptake. Furthermore, we found that MICU1 alternative splicing is induced during myogenesis by the splicing factor RBFOX2. These results highlight the complexity of the alternative splicing mechanisms in skeletal muscle and the regulation of mitochondrial Ca^2+^ among tissues.

## 1. Introduction

Alternative splicing is a process whereby different combinations of exons are spliced together to produce different mRNAs from a single pre-mRNA transcript, leading to the production of protein isoforms with diverse and even antagonistic functions. It is estimated to occur in 40–60% of all human transcripts, thereby underlying a major source for the diversity of the proteome [1,2,3]. The importance of alternative splicing is underlined by the fact that the majority of alternative exons are differentially expressed in developmental stages and tissues [4] and by the finding that mutations affecting alternative splicing are found in a large number of human diseases, ranging from cancer to neurological disorders [2,5,6].

Among tissues and skeletal muscle, together with the brain and heart, are the tissues that exhibit the highest levels of tissue-specific alternative splicing [7,8]. As for skeletal muscle, it is enriched in several muscle-specific and developmentally regulated splicing factors, such as FOX and Muscleblind-like (MBNL) families, suggesting that myogenesis is accompanied by high levels of alternative splicing regulation [9,10]. This mechanism ensures the expression of fiber-type specific isoforms of regulatory and contractile proteins during skeletal muscle myogenesis and in post-natal life to adapt muscular tissues to changes in metabolic and functional requirements [11,12]. Among the genes encoding for the contractile proteins, Tropomyosin and Troponin are well-studied paradigms, as they contain multiple alternative exons that are subjected to alternative splicing [13,14], thereby determining the kinetics of muscle contraction within muscle fibers [15,16].

Of note, many splicing factors are involved in the coordinated splicing of muscular proteins during myogenic differentiation, such as the polypyrimidine binding proteins (PTBP1 and PTBP2), quaking protein (QK), the Muscleblind-like family of proteins (MBNL1 and MBNL2), and the Fox homolog proteins (RBFOX1 and RBFOX2), just to name a few [17]. These splicing factors are also responsible for the expression of variants of the myogenic regulatory factors (MRFs), such as PAX7, hence regulating their activity during muscle development [18]. Importantly, many of these splicing factors are crucial in post-natal life. For example, it has been shown that skeletal muscle-specific RBFOX1 and RBFOX2 knockout in adult mice cause severe mass loss caused by increased protein degradation [19].

Alterations in the alternative splicing of muscle genes are implicated in the pathogenesis of several neuromuscular diseases [18,20]. In this context, reduced availability of the splicing factor MBNL1, due to the expression of expanded CUG or CCUG repeats in noncoding regions of the genes encoding the Myotonic Dystrophy Associated Protein Kinase (DMPK) and Zinc Finger Protein 9 (ZNF9), is responsible for Myotonic Dystrophy (DM) [21]. DM is the most common muscular dystrophy in adults and comprises two genetically distinct forms: DM type 1 (DM1) caused by an expansion of CTG repeats in the 3′-untranslated region (UTR) of the *DMPK* gene [22,23,24] and DM type 2 (DM2) caused by an expansion of CCTG repeats within the first intron of the *ZNF9* gene [25].

Moreover, the splicing alterations of muscle genes have been identified as secondary to muscle regeneration and cancer cachexia [26,27]. Other muscle pathologies caused or accompanied by alternative splicing defects are Pompe disease [28], myositis [29], and Duchenne Muscular Dystrophy [30].

Muscular alternative splicing mechanisms have been extensively shown to control Ca^2+^ homeostasis. Indeed, many genes involved in Ca^2+^ handling, and thus in the excitation–contraction coupling that determines the force of muscle contraction, are subjected to alternative splicing regulation [20]. Among these are the dihydropyridine receptors (DHPR), the L-type Ca^2+^ channel (Cav1.1) [31,32], ryanodine receptors (RyR) [33,34], and the SR Ca^2+^ adenosine triphosphatase (SERCA) [33,35]. Notably, aberrant splicing of genes involved in Ca^2+^ homeostasis has been observed in muscular dystrophy [33,36]. Recently, we have found a unique murine skeletal muscle-specific Mitochondrial Ca^2+^ Uniporter (MCU) Complex containing an alternative splicing isoform of the cooperative activator of the channel MICU1, MICU1.1, characterized by the inclusion of a highly conserved micro-exon encoding for four amino acids, which are sufficient to greatly alter the properties of the MCU [37]. Indeed, MICU1.1 binds Ca^2+^ with higher affinity than conventional MICU1 and, when heterodimerized with the gatekeeper of the channel MICU2, activates MCU current at lower Ca^2+^ concentrations than MICU1–MICU2 heterodimers [38]. This muscle-specific mechanism is required to sustain mitochondrial Ca^2+^ uptake and ATP production [38].

Here, we extended the analysis of MICU1 splicing to human tissues, revealing a higher complexity of splicing regulation of the MCU complex. Indeed, we found uncharacterized splicing variants that we analyzed for their ability to activate mitochondrial Ca^2+^ entry. Furthermore, we found that MICU1 splicing is regulated during myogenesis by the splicing factor RBFOX2.

## 2. Results

### 2.1. Characterization of the Human MICU1 Splice Variants

**Figure 1:**
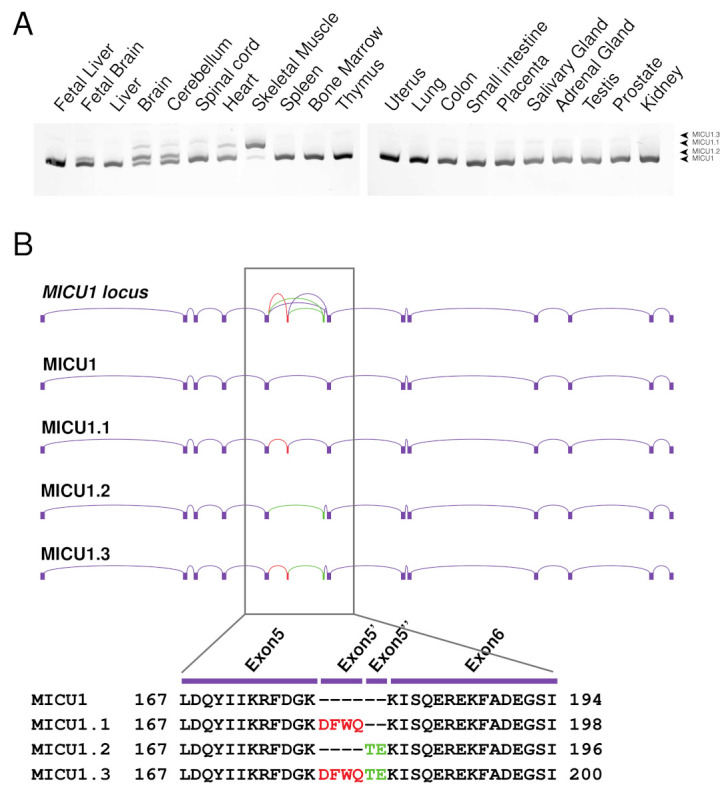
Alternative splicing of *MICU1*. (**A**) Representative acrylamide gel of PCR products using primers spanning the *MICU1* extra-exon of cDNA of different human tissues. *MICU1* produces a band of 102 bp, *MICU1.1* of 114 bp, *MICU1.2* of 108 bp and *MICU1.3* of 120 bp. (**B**) Schematic representation of the *Homo sapiens* MICU1 genomic locus (Chromosome 10: 72367340-72626131, reverse strand). In-frame inclusion of exon 5′ produces *MICU1.1*, of exon 5″ produces *MICU1.2,* and of both exon 5′ and exon 5″ produces *MICU1.3*. Alignment of the different resulting proteins is showed in the bottom panel. Red aminoacids indicate exon 5′, green exon 5′’.

We have previously shown that murine *MICU1* presents a skeletal muscle and brain-specific splice variant, which we named *MICU1.1* [38]. This variant is characterized by the addition of a micro-exon, exon 5′, encoding for four amino acids (EFWQ) inserted after amino acid 181 of the MICU1 sequence. To characterize *MICU1* splice variants in humans, we analyzed *Homo sapiens MICU1* splicing in a number of fetal and adult tissues mRNA by conventional PCR using primers spanning exon 5′ and separating the PCR products in an acrylamide gel (Figure 1A). The results show a complex scenario characterized by four major splice isoforms underlying the complexity of the tissue-specific regulation of mitochondrial Ca^2+^ uptake. DNA sequencing of these PCR bands shows the presence of conventional *MICU1*, the human isoform of *MICU1.1,* and another two splice variants, which we named *MICU1.2* and *MICU1.3* (Figure 1B). As observed in mice, *MICU1* is the prevalent isoform in most tissues and *MICU1.1* is the predominant isoform in skeletal muscle (Figure 1A). The sequence corresponding to the *MICU1.2* splice isoform is deposited in the NCBI database (NM_006077.4) and does not contain the 5′ extra-exon but a new exon, hereafter named 5″, which is composed of only two amino acids (TE) (Figure 1B). This variant is mostly expressed in neural tissues of the adult brain and cerebellum and in the fetal brain (Figure 1A). In addition, we found a fourth splice variant, which we named *MICU1.3* (NM_001363513.2), that contains both 5′ and 5″ exons (Figure 1A) and is predominantly expressed in the spinal cord and at lower levels in skeletal muscle.

### 2.2. Human MICU1 Splice Variants Display Different Effects on Mitochondrial Ca^2+^ Uptake

Next, we sought to analyze the effect of these human tissue-specific splicing variants of MICU1 on mitochondrial Ca^2+^ uptake.

**Figure 2:**
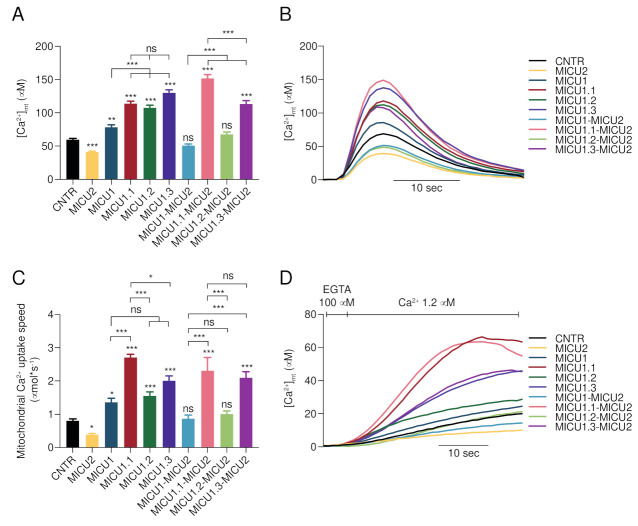
*MICU1* splicing variants differentially regulate the mitochondrial Ca^2+^ uptake. (**A**,**B**) [Ca^2+^]_mt_ measurements in intact HeLa cells transfected with the indicated constructs and challenged with maximal histamine stimulation. (**A**) Bar diagram representing the mean of the peak [Ca^2+^]_mt_. (**B**) Representative traces of the experiment. (*n* ≥ 25). (**C**,**D**) [Ca^2+^]_mt_ measurements in permeabilized HeLa cells transfected with the indicated constructs upon exposure to 1.2 µM [Ca^2+^]. (**C**) Bar diagram representing the mean [Ca^2+^]_mt_ speed. (**D**) Representative traces of the experiment. (*n* ≥ 5). Data are presented as mean ± SEM. One-way ANOVA was used with Tuckey tests for each sample. *ns* is non-significant, * *p* < 0.05, ** *p* < 0.01, and *** *p* < 0.005 compared with the specified conditions.

We have previously shown that overexpression of murine MICU1.1 in HeLa cells, which express only conventional MICU1, induces an increase of mitochondrial Ca^2+^ concentrations of about 25% compared with *MICU1*-overexpressing ones [38]. We thus cloned and overexpressed *Homo sapiens MICU1*, *MICU1.1*, *MICU1.2*, and *MICU1.3* in HeLa cells and measured the mitochondrial Ca^2+^ uptake in intact cells. As expected, *MICU1* overexpression causes a significant increase of mitochondrial Ca^2+^ uptake (Figure 2A,B). Consistent with the data on murine *MICU1.1*, human *MICU1.1*, when overexpressed alone, causes a higher increase of the [Ca^2+^]_mt_ peak compared with *MICU1* (45%; Figure 2A,B). Interestingly, similar effects were observed upon *MICU1.2* and *MICU1.3* overexpression (Figure 2A,B). Importantly, the different responses of the *MICU1* splice variants overexpression are not secondary to the different overexpression levels of the constructs (Figure S1).

We then decided to analyze the effect of the overexpression of the human splice variants on mitochondrial Ca^2+^ when co-expressed with the genuine MCU gatekeeper MICU2 [39]. As expected, *MICU2*-overexpressing HeLa cells showed a significant reduction in the [Ca^2+^]_mt_ peak and, in turn, its overexpression together with MICU1 leads to the blocking of the MICU1-dependent potentiation of the [Ca^2+^]_mt_ response (Figure 2A,B and [38,39]). Similar to what was observed with murine *MICU1.1*, *MICU2*, when overexpressed with human MICU1.1, is not able to inhibit *MICU1.1*-induced mitochondrial Ca^2+^ uptake increase (Figure 2A,B). The inclusion of the TE extra-exon in *MICU1.2* does not present the same behavior of *MICU1.1*. Indeed, mitochondrial Ca^2+^ uptake is indistinguishable in *MICU1*–*MICU2* and *MICU1.2*–*MICU2* overexpressing cells. As for MICU1.3, the MICU1.3–MICU2 heterodimer was able to increase the ability of mitochondria to take up Ca^2+^, although at a lower extent compared to the MICU1.1–MICU2 heterodimers.

We then evaluated the intrinsic mitochondrial Ca^2+^ uptake speed in the same conditions tested above in permeabilized HeLa cells, as previously performed [38]. After permeabilization on an EGTA-containing Ca^2+^-free intracellular buffer (IB/EGTA), Ca^2+^ accumulation was initiated by switching the perfusion buffer to IB, which contained an EGTA buffered [Ca^2+^] of 1.2 μM. As expected by the analysis with the mouse isoforms [38,39], *MICU1* and *MICU2* overexpressing cells show increased and decreased mitochondrial Ca^2+^ uptake speed, respectively (Figure 2C,D). An increase similar to that induced by *MICU1* overexpression is observed also in *MICU1.2* and *MICU1.3* overexpressing cells (Figure 2C,D). As for *MICU1.1*, similar to what we observed for mouse *MICU1.1* [38], its overexpression causes a strong increase of mitochondrial Ca^2+^ uptake speed, which was higher than that caused by conventional *MICU1* and the splice variants *MICU1.2* and *MICU1.3* (Figure 2C,D). Similar to the mitochondrial Ca^2+^ uptake measurements in intact cells described above, co-expression of *MICU1* together with *MICU2* blunts the increase in speed caused by the overexpression of *MICU1*, and the same is observed also for the *MICU1.2* splice variant. Strikingly, MICU2 is not able to inhibit the increase in the mitochondrial Ca^2+^ uptake speed, if co-expressed with *MICU1.1* or *MICU1.3* (Figure 2C,D).

### 2.3. MICU1 Introns Flanking 5′ Extra-Exon Present Multiple Splicing Factors Binding Sites

**Figure 3:**
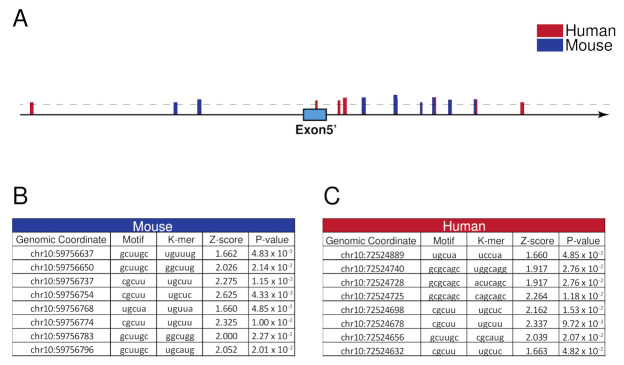
MBNL1 binding sites prediction in *Homo sapiens* and *Mus musculus* 300 bp surrounding the *MICU1.1* extra-exon. (**A**) Schematic representation of the predicted MBNL1 binding sites in proximity to the *MICU1.1* extra-exon. The predicted binding site in the human sequence is in red and the ones predicted in the mouse sequence are in blue. The dashed line represents the Z-score threshold, and the height of the band is proportional to the Z-score value. (**B**,**C**) Genomic coordinates, motives, K-mer, Z-score, and *p*-value of the identified binding sites. (**B**) MBNL1 binding sites in the mouse sequence. (**C**) MBNL1 binding sites in the human sequence.

We then focused our attention on the identification of the molecular mechanism regulating *MICU1.1* splicing. We thus analyzed the human and mouse genomic sequence flanking exon 5′ and assessed the presence of consensus sequences for splicing factors. Interestingly, several binding sites for MBNL1 were identified using the RBPmap3 bioinformatic tool ([40] and Figure 3A–C), suggesting a role for this splicing factor in the regulation of *MICU1* variants expression. Importantly, some predicted binding sites are conserved in the two species, supporting the hypothesis that MBNL1 could play a role in regulating the maturation of *MICU1* pre-mRNA.

### 2.4. MICU1 Alternative Splicing Events Do Not Require the Splicing Factor MBNL1

MBNL1 is a splicing factor associated with a genetic muscle disease, DM1. The expression of mutant RNAs containing hundreds to thousands of CUG or CCUG repeats interferes with the metabolism of other RNAs through the dysfunction of mainly two classes of RNA binding proteins. As a result, Muscleblind-like proteins (MBNL1, MBNL2, and MBNL3), which are RNA binding proteins specifically recognizing YGC RNA motifs, are titrated away from their normal mRNA targets as a result of their binding to expanded CUG and CCUG RNA repeats [41,42,43]. Of note, the splicing alterations affect several genes involved in Ca^2+^ signaling and ion transport, such as *RyR1*, *SERCA1*, *CACN1S,* and *CaV1.1* [44,45].

**Figure 4:**
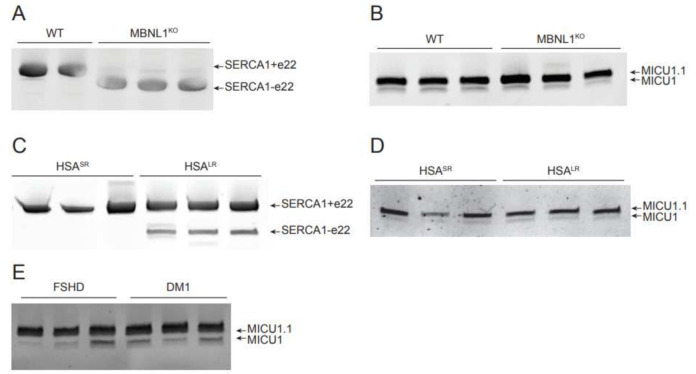
*MICU1* alternative splicing events do not require the splicing factor MBNL1. (**A**) Representative acrylamide gel of PCR products using primers spanning the *SERCA1* exon 22 of cDNA of WT or *MBNL1* KO mice. *SERCA1*+e22 produces a band of 218 bp, *SERCA1*-e22 produces a band of 176 bp. (**B**) Representative acrylamide gel of PCR products using primers spanning the *MICU1* extra exon of cDNA of WT or *MBNL1* KO mice. *MICU1* produces a band of 140 bp, *MICU1.1* of 152 bp. (**C**) Representative acrylamide gel of PCR products using primers spanning the *SERCA1* exon 22 of cDNA of *HSA*^SR^ or *HSA*^LR^ mice. *SERCA1*+e22 produces a band of 218 bp, *SERCA1*-e22 of 176 bp. (**D**) Representative acrylamide gel of PCR products using primers spanning the *MICU1* extra-exon of cDNA of *HSA*^SR^ or *HSA*^LR^ mice. *MICU1* produces a band of 140 bp, *MICU1.1* of 152 bp. (**E**) Representative acrylamide gel of PCR products using primers spanning the *MICU1* extra-exon of cDNA of human muscle biopsies from patients affected by FSHD or DM1. *MICU1* produces a band of 102 bp, *MICU1.1* of 114 bp.

To analyze whether MBNL1 controls *MICU1.1* splicing, we analyzed the effect of the removal of this splicing factor on *MICU1* transcript alternative splicing. To this end, we first analyzed human *MICU1* splicing events in the *MBNL1* knockout (KO) mouse, one of the disease models of DM [46]. We first analyzed the expression of the different *MICU1* alternative splicing variants in different types of murine muscles, since information on the distribution of the variants among muscles were not available. We found comparable expression of *MICU1* and *MICU1.1* among muscles and we decided to use gastrocnemius muscles for the subsequent analyses (Figure S2). We then verified that *MBNL1* KO muscles exhibit aberrant splicing of target mRNAs. To this end, we chose SERCA1, which presents two alternative splicing variants. The alternative splicing of exon 22 affects the translation of seven amino acids located at the C-terminal of the SERCA1 protein and it has been shown, both in mouse models of DM and in patients, that the lack of MBNL1 prevents the inclusion of exon 22 in the mature mRNA [21,33,47,48]. As expected, in *MBNL1* KO muscles the predominant isoform expressed is the SERCA1 without exon 22 (Figure 4A). We then analyzed the alternative splicing isoform expression of *MICU1* in the *MBNL1* KO gastrocnemius muscle, finding *MICU1.1* as the predominant isoform in the control muscles (Figure 4B). We also analyzed another model of MBNL1 deficiency, the *HSA*^LR^ transgenic mouse model. This transgenic mouse carries a human skeletal α-actin (*HSA*) gene modified by the insertion of either 5 (*HSA* short repeat, *HSA*^SR^) or 250 (*HSA* long repeat, *HSA*^LR^) CTG repeats in the *HSA* 3′UTR [49]. The *HSA*^SR^ mice carrying repeats in the normal range are indistinguishable from the control. In contrast, *HSA*^LR^ mice develop severe myotonia and dystrophic muscle features of DM. Also in this model, as expected, aberrant *SERCA1* splicing is observed (Figure 4C and [21,33]) and, similar to the *MBNL1* KO model, *MICU1* splicing was not affected (Figure 4D). We finally analyzed *MICU1* alternative splicing events in patients affected by DM1 and we compared MICU1 alternative splicing events with those of muscles of patients affected by facioscapulohumeral muscular dystrophy (FSHD), a pathological condition where splicing is not affected [50]. Similar to the two DM mouse models, *MICU1* splicing regulation in this disease is indistinguishable from what was observed in muscles from FSHD patients (Figure 4E).

### 2.5. MICU1 Alterative Splicing Is Regulated during Embryonic Development by the Splicing Factor RBFOX2

**Figure 5:**
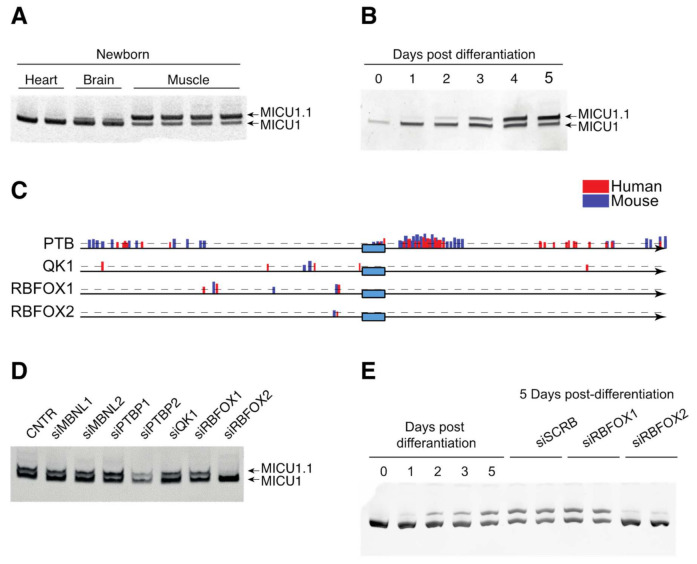
MICU1 alterative splicing is regulated by the splicing factor RBFOX2 during embryonic development. (**A**) Representative acrylamide gel of PCR products using primers spanning the *MICU1* extra-exon of cDNA of newborn brain, heart, and muscle. *MICU1* produces a band of 140 bp, *MICU1.1* of 152 bp. (**B**) Representative acrylamide gel of PCR products using primers spanning the *MICU1* extra-exon of cDNA of C2C12 at day 0, 1, 2, 3, 4, or 5 of differentiation. *MICU1* produces a band of 140, *MICU1.1* of 152 bp. (**C**) Schematic representation of the predicted PTB, QK1, RBFOX1, and RBFOX2 binding sites in proximity of the *MICU1.1* extra-exon. The predicted binding sites in the human sequence are in red and the ones predicted in the mouse sequence are in blue. The dashed line represents the Z-score threshold, and the height of the band is proportional to the Z-score value. (**D**) Representative acrylamide gel of PCR products using primers spanning the *MICU1* extra-exon of cDNA of C2C12 transfected at day 3 of differentiation with the indicated siRNA. *MICU1* produces a band of 140 bp, *MICU1.1* of 152 bp. (**E**) Representative acrylamide gel of PCR products using primers spanning the *MICU1* extra-exon of cDNA of C2C12 transfected at day 3 of differentiation with siRNA against RBFOX1 and RBFOX2. *MICU1* produces a band of 140 bp, *MICU1.1* of 152 bp.

We then decided to analyze the *MICU1* alternative splicing events during skeletal muscle development. To this end, we first analyzed *MICU1* splice variants’ expression in heart, brain, and skeletal muscle from newborn mice. As shown in Figure 5A, newborn muscles co-express both conventional *MICU1* and *MICU1.1*, suggesting that the predominant expression of *MICU1.1* in adult muscles is caused by a regulation event either in the late stages of embryonic development or in the early post-natal life. To confirm this hypothesis, we analyzed the expression of the *MICU1* alternative splicing variants during the progression of C2C12 myoblast differentiation to myotubes *in vitro*. Myoblasts only express conventional *MICU1*, while, upon differentiation, *MICU1.1* starts to accumulate (Figure 5B). To uncover the mechanism of this regulation, we searched the factors regulating alternative splicing during skeletal muscle embryonic and post-natal development and we selected MBNL1, MBNL2, Polypyrimidine Tract Binding Protein 1 (PTBP1) and 2 (PTBP2), Quaking 1 (QK1), RNA Binding Fox-1 Homolog 1 (RBFOX1) and 2 (RBFOX2), which have been extensively shown to regulate splicing during muscle and C2C12 differentiation [17,51,52]. We first decided to analyze the human and mouse genomic sequence flanking *MICU1* exon 5′ and assessed the presence of consensus sequences for the binding of these splicing factors. Interestingly, several binding sites for PTBP, QK1, RBFOX1, and RBFOX2 were identified (Figure 5C and Figure S3), suggesting a possible role for these splicing factors in the regulation of *MICU1* variants expression. Thus, we decided to silence each of them in C2C12 and tested the silencing efficiencies (Figure S4). Strikingly, RBFOX2 silencing was able to completely block the generation of the *MICU1.1* splice isoform (Figure 5D). Thus, we focused our attention on this splicing factor by analyzing the effect of its silencing on the splicing events during C2C12 differentiation. To this end, we transfected C2C12 myoblasts with specific siRNAs for RBFOX2 and RBFOX1 as control for siRNA specificity. We then differentiated myoblasts in myotubes and analyzed MICU1 splicing isoforms at five days post differentiation. Only the silencing of RBFOX2 was able to inhibit MICU1 alternative splicing (Figure 5E).

## 3. Discussion

Alternative splicing is modulated in a tissue- and developmental stage-specific manner and the regulation of this process is essential for diverse cellular functions in both physiological and pathological situations [53,54]. This process is particularly important in skeletal muscle that, together with the brain and heart, is the tissue that exhibits the highest levels of tissue-specific and conserved alternative splicing [7,8,55,56]. Nevertheless, how alternative splicing is regulated to adapt skeletal muscle isoform expression and sarcomere mechanics and metabolism has remained elusive. Many proteins essential for striated muscle development exist in multiple isoforms generated by alternative splicing. These include myogenic transcription factors, metabolic enzymes and components of the myofibril [20,56]. In this regard, we recently discovered that skeletal muscle mitochondrial Ca^2+^ uptake is regulated by alternative splicing mechanisms. Indeed, we found that skeletal muscle expresses a muscle-specific alternative splicing isoform of the MCU regulator MICU1, which is characterized by the addition of a micro-exon of four amino acids that is sufficient to greatly modify the properties of the MCU [38]. Indeed, MICU1.1 binds Ca^2+^ one order of magnitude more efficiently than MICU1 and, when heterodimerized with MICU2, activates MCU current at lower Ca^2+^ concentrations than MICU1–MICU2 heterodimers [38]. As for the physiological role of this variant, we showed that MICU1.1 is required in muscle where fast Ca^2+^ transients occur to ensure sustained ATP production during resistance and strenuous exercise [38].

Here, we deepened the characterization of *MICU1* alternative splicing mechanisms by studying its splicing in *Homo sapiens*. The analysis of alternative splicing events between exon 5 and exon 6 of *MICU1* in several fetal and adult tissues underlined a complex scenario (Figure 1A). Interestingly, while most tissues express conventional MICU1, tissues characterized by the highest levels of alternative splicing events, i.e., skeletal muscle, brain, and heart, present the concomitant expression of multiple variants. As expected by the study of murine MICU1 splicing regulation [38], human skeletal muscle also mostly expresses MICU1.1. The cloning and sequencing of the splicing variants in brain, heart, and skeletal muscle revealed the existence of two extra-exons, exon 5′ characterized by four amino acids—similar to that of mouse MICU1.1—and exon 5″ characterized by two amino acids, leading to the generation of four splicing variants (Figure 1B) and highlighting the complexity of mitochondrial Ca^2+^ uptake in this tissue. Ca^2+^ measurements demonstrated that the addition of exon 5″ alone does not alter the properties of MICU1-mediated activation of MCU, with or without MICU2—both in intact and permeabilized HeLa cells (Figure 2A–D). Intriguingly, both isoforms including exon 5′ with (MICU1.3) or without (MICU1) exon 5″ share the same regulatory properties on mitochondrial Ca^2+^ uptake (Figure 2A–D). As well, the addition of exon 5″ lowers the synergistic activation of MCU when co-expressed with MICU2. As in *Mus musculus*, the clarification of the structural role of this extra-exon’s insertion is not possible since they are far from the Ca^2+^-binding EF-hand sites, and the numerous crystal structure models of MICU1 did not resolve the loop region that contains the extra-exons [57,58,59,60,61,62,63,64], indicating that this region is flexible and thus not visible in the electron density map. Therefore, for human MICU1.1 and MICU1.3, we cannot infer on the mechanism by which the extra-exon modifies the affinity of the EF-hand domains and the interaction with MICU2. Importantly, human MICU1.1-dependent regulation of MCU resembles that of murine MICU1.1 [38]. Indeed, as murine MICU1.1, the human isoform activates MCU at a higher extent than conventional MICU1 and MICU2 is not able to decrease the MICU1.1-dependent potentiation of [Ca^2+^]_mt_ (Figure 2A–D and [38]). Interestingly, the splice variant characterized by the addition of both exon 5′ and 5″, which is indistinguishable from MICU1.1 with respect to the regulation of mitochondrial Ca^2+^, is expressed in the central nervous system (Figure 1A). Notably, the brain, and, to a lesser extent, skeletal muscle, express a novel vertebrate paralog of *MICU1*, *MICU3*, which likely arose from a gene duplication event prior to vertebrate evolution [65]. In neurons, we recently showed that the role of MICU3 is to enhance MCU opening, thereby forming a disulfide bond-mediated dimer with MICU1, but not with MICU2 [65]. Coherently, the crystal structure of MICU3 revealed that the overall structures of MICU2 and MICU3 are extremely similar in the N-terminal region [66]. As for the pathophysiological role, silencing of MICU3 in primary cortical neurons impairs Ca^2+^ signals elicited by synaptic activity, thus suggesting a specific role in regulating neuronal function by coordinating ATP synthesis and consumption in firing nerve terminals [65,67]. This was confirmed by the neurological impairment of *Drosophila melanogaster* lacking MICU3 [68]. Furthermore, MICU3 expression was inhibited in skeletal muscle during aging and its silencing is associated with decreased myogenesis but increased oxidative stress and apoptosis [69], suggesting that this isoform might be essential to attenuate oxidative stress and apoptosis and to restore skeletal muscle mass and function.

We also searched the splicing factors responsible for *MICU1* alternative splicing events. Bioinformatic analysis of the introns flanking exons 5′ and 5″ revealed the presence of MBNL1 splice acceptor and donor sites. Despite this, the analysis of *MICU1* alternative splicing events in both the *MBNL1* KO and *HSA*^LR^ animals—which are models of DM [46,49]—as well as in patients affected by DM, clearly demonstrated that MBNL1 does not control *MICU1* splicing events (Figure 4). We then changed our approach and searched for processes in which *MICU1* alternative splicing was regulated. We first analyzed newborn heart, brain, and skeletal muscles and we found that, while the brain and cardiac alternative splicing variants expression is indistinguishable from that of adult muscles, the skeletal muscle of newborn mice co-expresses an almost equal amount of *MICU1* and *MICU1.1*, suggesting that *MICU1* alternative splicing might be regulated during muscle development (Figure 5A). This was confirmed by analysis of C2C12 differentiation that showed that, at the myoblast stage, conventional *MICU1* is the only isoform present and, during the course of differentiation, *MICU1* expression progressively decreased correspondingly to increased expression of *MICU1.1* (Figure 5B). Thus, we analyzed *MICU1* alternative splicing events upon silencing of splicing factors involved in skeletal muscle differentiation. For this purpose, we analyzed MBNL1 and 2, PTB1 and 2, QK1, and RBFOX1 and 2, which have been shown to regulate muscle-specific alternative splicing events during muscle development [17,51,52], and several putative binding sites of these splicing factors were predicted in close proximity to the 5′ exon of *MICU1.1* (Figure 5C and Figure S3). It has long been known that the functional diversity of skeletal muscle cells is reflected by the wide array of protein isoforms that are found in differentiating, developing, and mature muscle fibers, which allows skeletal muscle to perform a very diverse array of functions throughout the body [70]. This is determined by either the expression of specific members of multigene families (e.g., heavy chain subunit of myosin) or by alternate RNA processing from a single gene (e.g., troponin T subunits) coding for muscle functional and structural proteins that ensures the heterogeneity in the types of myofibers that differ in their contractile ability and metabolic requirements [70]. Recent studies confirmed that alternative splicing contributes to muscle development, and misregulation of RNA processing is implicated in muscle diseases [71]. Our data suggest that skeletal muscle alternative splicing is not limited to structural proteins but also to mitochondrial Ca^2+^ homeostasis. Indeed, here we demonstrate that MICU1 alternative splicing is specifically controlled by the splicing factor RBFOX2 (Figure 5D,E), which has been shown to be required for muscle development and differentiation [19,52,72,73]. Importantly, the RBFOX family of RNA-binding proteins contains three genes: *RBFOX1*, *RBFOX2,* and *RBFOX3*. While *RBFOX3* expression is restricted to neurons, *RBFOX2* is widely expressed in whole embryos, ovaries, stem cells, the brain, skeletal muscles, and the heart, and *RBFOX1* is selectively expressed in brain, heart and skeletal muscle [74]. The expression pattern of *RBFOX2* is not compatible with the prevalence of *MICU1.1* splicing in skeletal muscle. Nevertheless, we cannot exclude the fact that this effect may depend on the varying abundance, localization, and phosphorylation of this protein. In support of this notion, *RBFOX2* is upregulated in the late stages of terminal muscle differentiation [20,75] and the RBFOX2 transcript contains two exons that undergo regulated skeletal muscle-specific splicing changes, which could alter its activity independently from its steady-state levels [52]. In addition, splicing events are controlled by an extensive range of proteins playing multiple regulatory roles within the cell [76]. The mechanisms of these proteins span a wide range and include the recruitment of spliceosomal components, blocking access to splice sites, and altering the spatial proximity between exons [77]. As well, splicing of a given mRNA is also affected by the epigenetic environment of the gene locus [76]. Furthermore, the possibility that different splicing factors can modulate in the opposite direction of the splicing of a specific exon in different tissues can conciliate the absence of MICU1.1 splicing in tissues in which RBFOX2 is expressed. In this scenario, the absence of a splicing factor blocking access to the splice sites of exon 5′ can explain the tissue-specific expression of MICU1.1 in skeletal muscle, while in other tissues, which also express RBFOX2, the maturation of MICU1 pre-mRNA towards MICU1.1 is prevented.

Importantly, recent data showed that RBFOX2 depletion prevents myoblast fusion partially through splicing regulation of the transcription factor known as myocyte-specific enhancer factor-2D (Mef2d) [73,75]. Furthermore, deletion of both *RBFOX1* and *RBFOX2* in mice resulted in a rapid and severe loss of muscle mass and alteration of splicing of numerous transcripts [19]. Since we found that mitochondrial Ca^2+^ positively regulates skeletal muscle trophism [78], the splicing alteration of the *MICU1* gene could contribute to the phenotype of *RBFOX2* KO mice. Moreover, the clarification of the roles of these specific skeletal-muscle isoforms represents an important task for the future, with potential pharmacological applications to treat muscle loss in disease states and in aging.

## 4. Materials and Methods

### 4.1. Mice

Adult male *MBNL1* WT, *MBNL1* KO, *HSA*^SR^, and *HSA*^LR^ mice (2–3 months old) were used for all the experiments. The *MBNL1* KO mice were generated in the laboratory of Prof. Swanson [46]. The heterozygous *MBNL1* WT/KO mice were cross-bred to obtain the control and the full-KO mice for the experiments. The muscles of the *HSA*^SR^ and *HSA*^LR^ mice were kindly provided by the laboratory of Prof. Thornton [49].

All mice were housed in a temperature-controlled facility under a 12-h light/dark cycle with standard environmental enrichment and ad libitum access to standard rodent chow and water. All experiments were conducted with male mice that were 8–12 weeks old. The study was conducted according to the guidelines of the Declaration of Helsinki and approved by the Animal Care Office at the University of Padova and performed in accordance with the Italian law D. L. n.26/2014.

### 4.2. Cell Culture and Transfection

HeLa and C2C12 cells were cultured in DMEM (Thermo Fisher Scientific, Monza MB, Italy) supplemented with 10% FBS (Thermo Fisher Scientific, Monza MB, Italy) and containing penicillin (100 U/mL) and streptomycin (100 mg/mL). HeLa cells were transfected with a standard Ca^2+^-phosphate procedure as already performed [79,80]. All experiments were carried out 24–36 h after transfection. Mock vectors (i.e., pcDNA3.1) were used as controls in all overexpression experiments (referred to as the control condition). C2C12 differentiation in myotubes was obtained through the changing media at 90% of confluences and incubation in DMEM supplemented with 2% Horse Serum (Thermo Fisher Scientific, Monza MB, Italy) for 5 days. For silencing experiments, C2C12 cells at 3 days post-differentiation were transfected with RNAiMax (Thermo Fisher Scientific, Monza MB, Italy) following manufacturer instructions. A non-targeting siRNA (i.e., siRNA-scrambled) was used as a control in all the silencing experiments.

### 4.3. Aequorin Ca^2+^ Measurements

HeLa cells grown on 13-mm round glass coverslips at 60% confluence in a 24–well plate were transfected with the indicated constructs and the low-affinity mitochondrial (mtAEQmut) probe with the Ca^2+^-phosphate procedure with the appropriate mix of DNA (in an aequorin/total DNA ratio of 1:4), as described previously [81]. Ca^2+^ measurements were performed as described previously [39]. Output data were analyzed and calibrated with a custom-made macro-enabled Excel workbook.

The experiments with permeabilized cells were performed as described previously [39]. Mitochondrial Ca^2+^ uptake speed was calculated as the first derivative by using the SLOPE Excel function and smoothed for three time points. The higher value reached during Ca^2+^ addition represents the maximal Ca^2+^ uptake speed. All materials were from Merck unless specified otherwise.

### 4.4. RNA Extraction, Reverse Transcription, PCR and qPCR

For cDNA preparation from C2C12 cells, the total RNA was extracted using a TRIZOL reagent (Thermo Fisher Scientific, Monza MB, Italy) and following manufacturer instructions. For the human samples, a human mRNA library was used (Clontech, Saint-Germain-en-Laye France) and retro-transcribed as follows.

The RNA was quantified with Nanodrop (Thermo Fisher Scientific, Monza MB, Italy) and 1 μg of total RNA of each sample was retro-transcribed using the cDNA synthesis kit SuperScript II (Thermo Fisher Scientific, Monza MB, Italy). Oligo(dT)_12–18_ primer (Thermo Fisher Scientific, Monza MB, Italy) was used as a primer for first stand cDNA synthesis with reverse transcriptase.

The obtained cDNAs were analyzed by PCR with primers that localized in exon 5 and exon 6 of *MICU1* to highlight the splicing junction between these two exons, as previously performed [38].

For Homo sapiens *MICU1*:

Fw: 5′-CTTGGGTCTGGATCAATA-3′

Rv: 5′-CAAGGGTGTAAAATATACTG-3′

Product size: 102 bp for *MICU1*, 114 bp for *MICU1.1*, 108 bp for *MICU1.2*, and 120 bp for *MICU1.3*.

For Mus musculus *MICU1*:

Fw: 5′-GAACACTTGGGCCTGGATCA-3′

Rv: 5′-GAAGGAGATGAGCCCACACT-3′

Product size 152 bp for *MICU1.1* and 140 bp for *MICU1*.

For Mus musculus *SERCA1* exon 22 retention (*SERCA1*-e22):

Fw: 5′-GCTCATGGTCCTCAAGATCTCAC-3′

Rv: 5′-GGGTCAGTGCCTCAGCTTTG-3′

Product size 218 bp for *SERCA1*+e22 and 176 bp for *SERCA1*-e22.

The specific bands were amplified with the Q5 High-Fidelity DNA Polymerase (NEB, Ipswich, MA, USA) and separated in 10% acrylamide gels in TBE. The bands were visualized with the fluorescent dye Gel Red Nucleic Acid Stain (Biotium, Bergamo, Italy) and visualized with a UV transilluminator.

For the qPCR experiment, cDNA obtained from C2C12 myoblasts transfected with either a control scramble or siRNA designed to silence splicing factors involved in myogenic differentiation was analyzed using the IQ5 thermocycler and SYBR green chemistry (Bio-Rad, Segrate MI, Italy). The primers were designed and analyzed with Primer 3 (http://bioinfo.ut.ee/primer3-0.4.0/, accessed on 10 April 2017) and their efficiency was between 95% and 100%. The housekeeping genes *GAPDH* were used for cDNA normalization. For quantification, the expression levels were calculated by using the 2^-ΔΔCT^ Method [82].

The following oligonucleotide primers were used:

*GAPDH:*

fw: 5′-CACCATCTTCCAGGAGCGAG-3′

rv: 5′-ACAGTTCCGAGCGTCAAAGACC-3′

*MBNL1:*

Fw: 5′-CACTGGCAGTTCTAGTGTGGA-3′

Rv: 5′-ATGGGTTCCAACCCGTAATGT-3′

*MBNL2:*

Fw: 5′-ACAGCTCCGGTAGTTAGGGA-3′

Rv: 5′-TGACCAGTGGTGTATGGCTG-3′

*PTBP1:*

Fw: 5′-CCGAGCCTCATTGTGACCTT-3′

Rv: 5′-GTCACTGGAAGGAGCTCAGG-3′

*PTBP2:*

Fw: 5′-CACACTCTTGTGGGACGTGT-3′

Rv: 5′-GCACATGCTCAGAAATCCACA-3′

*QK1:*

Fw: 5′-TGAGTAATGCTGGCTCTGAACA-3′

Rv: 5′-ACTCCCATCTACCCACGCTA-3′

*RBFOX1:*

Fw: 5′-ACATCACGTTCCCACTTCCC-3′

Rv: 5′-TGCAGGTTCTGGGCTTGAAA-3′

*RBFOX2:*

Fw: 5′-GGCACGCGAGTTTTTGAGTT-3′

Rv: 5′-CAACAAGCACGCACACAAGA-3′

### 4.5. siRNAs and Constructs

For silencing of the specific splicing factors in the C2C12 cell lines, the following siRNA were used (Merck):

*MBNL1*: EMU009071

*MBNL2*: EMU081401

*RBFOX1*: SASI_Mm01_00150775

*RBFOX2*: EMU169371

PTBP1: EMU219241

PTBP2: EMU054891

QKI: EMNC001731

Human *MICU1*, *MICU1.1*, *MICU1.2*, and *MICU1.3* were amplified from human skeletal muscle cDNA (Clontech). For the cloning of the HA-tagged version of *MICU1.1*, *MICU1.2,* and *MICU1.3* in pcDNA3.1:

fw: 5′-CGGATCCGCCACC ATGTTTCGTCTGAACTCACT-3′

rv: 5′-AATTCTCGAGTCACAGGGAAGCGTAGTCAGGCACATCGTAGGGGTACTGTTTGGGTAAAGCGAAGT-3′

The PCR fragment was cloned into BamHI and XhoI sites in pcDNA3.1 (Thermo Fisher Scientific, Monza MB, Italy).

### 4.6. Western Blotting

To monitor overexpressed proteins, cells were lysated in RIPA-buffer (150 mM NaCl, 50 mM Tris, 1 mM EGTA, 1% Triton X-100, 0.1% SDS) and, after 30 min of incubation on ice and centrifugation at 15,000× *g* for 10 min to remove debris, 40 μg of total proteins were loaded, according to BCA quantification (Thermo Fisher Scientific, Monza MB, Italy). The proteins were reduced with 100 mM DTT and denatured for 5 min at 90 °C. The proteins were then separated by SDS-PAGE electrophoresis, in 4–12% acrylamide gels (Thermo Fisher Scientific, Monza MB, Italy) and transferred on nitrocellulose membranes (Thermo Fisher Scientific, Monza MB, Italy) by wet electrophoretic transfer. The blots were blocked for 1 h at RT with 5% non-fat dry milk (Bio-Rad, Segrate MI, Italy) in TBS-Tween (0.5 M Tris, 1.5 M NaCl, 0.01% Tween) solution and incubated overnight at 4 °C with primary antibodies. Horseradish peroxidase-conjugated secondary antibodies (Bio-Rad, Segrate MI, Italy) were incubated for 1 h at room temperature, followed by detection by chemioluminescence (SuperSignal Pico, Thermo Fisher Scientific, Monza MB, Italy). The following antibodies were used: α-HA (1:1000, Cell Signaling Technologies, catalogue number 3724) and α-TOM20 (Santa Cruz biotechnology, Dallas, TX, USA). Secondary, isotype-specific HRP-conjugated antibodies (1:5000) were purchased from Bio-Rad (Segrate MI, Italy). The western blots shown in the figures are representative of at least three different independent experiments.

### 4.7. Prediction of Splicing Factors Binding Sites

To predict the binding site of MBNL1 to MICU1 pre-mRNA, the online available tool RBPmap3 was used [40]. In detail, RBPmap was interrogated with the genomic sequence of *MICU1*, 150 bp upstream and downstream of the *MICU1.1* extra-exon. The following genomic coordinates were used:

*Homo sapiens* (assembly hg38) chr10:72524585-72524896:-

*Mus musculus* (assembly mm10) chr10:59756556-59756867:+

The tool was run with default parameters (default stringency level and without conservation filter) for the motifs recognized by MBNL1 (GCUUGC, CGCUU, GCGCAGC, UGCUA), PTB (CUUUCU, UCUU, CUCUCU), QK1 (ACUAAY), RBFOX1 (WGCAUGM), and RBFOX2(GCAUG). The results obtained were overlaid in Figure 3A and Figure 5C and the statistical results were reported in the table in Figure 3B and Figure S3.

### 4.8. Statistical Analysis of Data

All data were analyzed for statistical significance using GraphPad Prism software. All data are expressed as mean ± SD unless otherwise specified. For comparison between two independent groups, unpaired Student’s t tests were used. For comparison between three or more independent groups, one-way ANOVA was used with post hoc Bonferroni tests for each sample. Adjusted * *p* < 0.05, ** *p* < 0.01, *** *p* < 0.005, and **** *p*<0.001.

## Data Availability

Not applicable.

## Supplementary Materials

The following supporting information can be downloaded at: https://www.mdpi.com/article/10.3390/ijms23052517/s1.
