# Supplementary material for: The Splicing of the Mitochondrial Calcium Uniporter Genuine Activator MICU1 Is Driven by RBFOX2 Splicing Factor during Myogenic Differentiation
Source: Int J Mol Sci. 2022 Feb 24;23(5):2517. doi: 10.3390/ijms23052517 (PMC8909990; doi:10.3390/ijms23052517)
Supplement: Supplementary file 1 [file ijms-23-02517-s001.zip › ijms-1589987-supplementary.pdf]

International Journal of *Molecular Sciences*

MDPI

Article

The Splicing of the Mitochondrial Calcium Uniporter Genuine Activator MICU1 Is Driven by RBFOX2 Splicing Factor during Myogenic Differentiation

Denis Vecellio Reane [1], Cristina Cerqua [3,4], Sabrina Sacconi [5], Leonardo Salviati [2,3,4], Eva Trevisson [3,4,*] and Anna Raffaello [1,2,*]

1 Department of Biomedical Sciences, University of Padova, 35131 Padova, Italy; denis.vecellioreane@unipd.it
2 Myology Center, University of Padova, 35131 Padova, Italy; leonardo.salviati@unipd.it
3 Clinical Genetics Unit, Department of Women's and Children's Health, University of Padova, Via Giustiniani 3, 35128 Padova, Italy; cristina.cerqua@unipd.it
4 Istituto di Ricerca Pediatrica—IRP, Fondazione Città della Speranza, 35127 Padova, Italy
5 Centre Hospitalier Universitaire de Nice, Peripheral Nervous System and Muscle Department, Rare Neuromuscular Disease Reference Center, ERN-Euro-NMD, Université Côte d'Azur (UCA), 06103 Nice, France; sacconi@unice.fr
* Correspondence: eva.trevisson@unipd.it (E.T.); anna.raffaello@unipd.it (A.R.)

Supplementary Materials:

Citation: Vecellio Reane, D.; Cerqua, V.; Sacconi, S.; Salviati, L.; Trevisson, E.; Raffaello, A. The Splicing of the Mitochondrial Calcium Uniporter Genuine Activator MICU1 Is Driven by RBFOX2 Splicing Factor during Myogenic Differentiation. *Int. J. Mol. Sci.* **2022**, *23*, 2517. https://doi.org/10.3390/ijms23052517

Academic Editor: Daniel Taillandier

Received: 24 January 2022
Accepted: 22 February 2022
Published: 24 February 2022

Publisher's Note: MDPI stays neutral with regard to jurisdictional claims in published maps and institutional affiliations.

Copyright: © 2022 by the authors. Licensee MDPI, Basel, Switzerland. This article is an open access article distributed under the terms and conditions of the Creative Commons Attribution (CC BY) license (http://creativecommons.org/licenses/by/4.0/).

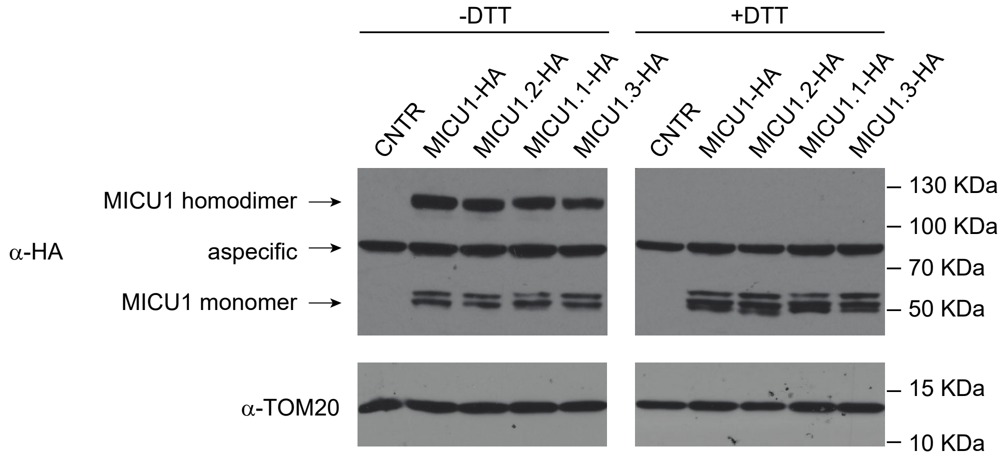

Figure S1. Monomer, homodimer formation and protein expression levels of human MICU1 splicing variants**.** HeLa cells were harvested after 24 hours of transfection with the indicated constructs, and total protein was extracted and subjected to western blotting analysis with α-HA antibody. SDS-PAGE was performed both in the presence and in the absence of DTT. *α*-TOM20 was used as loading control.

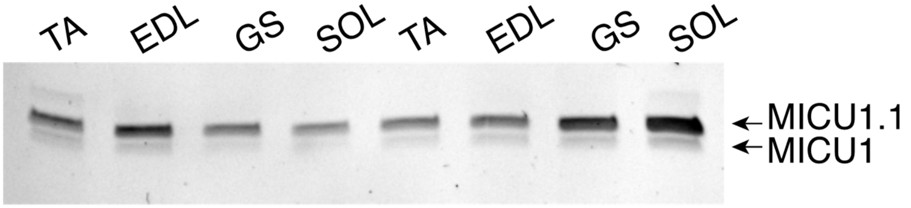

Figure S2. MICU1 and MICU1.1 alternative splicing regulation among different mouse muscles**.** Representative acrylamide gel of PCR products using primers spanning the *MICU1* extra exon of cDNA of mouse muscles (TA: *Tibialis anterior*; EDL: *Extensor digitorum longus muscle*; GS: *Gastrocnemius* and SOL: *soleus*). *MICU1* produces a band of 140 bp, *MICU1.1* of 152 bp.

A

Mouse					
Genomic Coordinate	Motif	K-mer	Z-score	P-value	
chr10:59756556	cuuucu	ucuucu	2.309	1.05×10^{-2}	PTB
chr10:59756556	ucuu	ucuu	3.259	5.59×10^{-4}	
chr10:59756559	ucuu	ucuu	3.268	5.42×10^{-4}	
chr10:59756564	ucuu	uauu	2.429	7.57×10^{-3}	
chr10:59756566	cuuucu	uuuuca	2.691	3.56×10^{-3}	
chr10:59756566	ucuu	uuuu	2.429	7.57×10^{-3}	
chr10:59756584	ucuu	ucau	2.152	1.57×10^{-2}	
chr10:59756587	cuuucu	uuuuca	1.679	4.66×10^{-2}	
chr10:59756664	cucucu	aucacu	2.58	4.94×10^{-3}	
chr10:59756666	cucucu	cacugu	2.625	4.33×10^{-3}	
chr10:59756668	cuuucu	cugucg	2.914	1.78×10^{-3}	
chr10:59756668	cucucu	cugucg	2.625	4.33×10^{-3}	
chr10:59756670	cucucu	gucgau	1.884	2.98×10^{-2}	
chr10:59756672	cucucu	cgauuu	1.884	2.98×10^{-2}	
chr10:59756674	cuuucu	auuugu	2.938	1.65×10^{-3}	
chr10:59756674	cucucu	auuugu	1.884	2.98×10^{-2}	
chr10:59756676	cucucu	uuguuu	1.884	2.98×10^{-2}	
chr10:59756677	ucuu	uguu	2.152	1.57×10^{-2}	
chr10:59756678	cuuucu	guuuuu	2.938	1.65×10^{-3}	
chr10:59756678	cucucu	guuuuu	1.884	2.98×10^{-2}	
chr10:59756679	ucuu	uuuu	2.152	1.57×10^{-2}	
chr10:59756680	cuuucu	uuuucu	3.728	9.65×10^{-5}	
chr10:59756680	ucuu	uuuu	2.152	1.57×10^{-2}	
chr10:59756680	cucucu	uuuucu	2.625	4.33×10^{-3}	
chr10:59756682	cucucu	uucuuu	2.625	4.33×10^{-3}	
chr10:59756683	ucuu	ucuu	2.714	3.32×10^{-3}	
chr10:59756684	cuuucu	cuuucu	3.877	5.29×10^{-5}	
chr10:59756684	cucucu	cuuucu	3.33	4.34×10^{-4}	
chr10:59756686	cuuucu	uucucu	3.309	4.68×10^{-4}	
chr10:59756686	cucucu	uucucu	3.33	4.34×10^{-4}	
chr10:59756687	ucuu	ucuc	2.152	1.57×10^{-2}	
chr10:59756688	cuuucu	cucucu	3.568	1.80×10^{-4}	
chr10:59756688	cucucu	cucucu	3.696	1.10×10^{-4}	
chr10:59756689	ucuu	ucuc	2.152	1.57×10^{-2}	
chr10:59756690	cuuucu	cucucc	3.074	1.06×10^{-3}	
chr10:59756690	cucucu	cucucc	3.321	4.48×10^{-4}	
chr10:59756691	ucuu	ucuc	2.152	1.57×10^{-2}	
chr10:59756692	cucucu	cuccuu	2.625	4.33×10^{-3}	
chr10:59756693	ucuu	uccu	2.152	1.57×10^{-2}	
chr10:59756694	cuuucu	ccuucu	3.407	3.28×10^{-4}	
chr10:59756694	ucuu	ccuu	2.152	1.57×10^{-2}	
chr10:59756694	cucucu	ccuucu	2.625	4.33×10^{-3}	
chr10:59756696	cucucu	uucuga	1.884	2.98×10^{-2}	
chr10:59756697	ucuu	ucug	2.152	1.57×10^{-2}	
chr10:59756707	cucucu	aguucu	1.884	2.98×10^{-2}	
chr10:59756709	cucucu	uucugg	1.875	3.04×10^{-2}	
chr10:59756711	cucucu	cuggca	1.821	3.43×10^{-2}	
chr10:59756801	cucucu	gccucu	2.027	2.13×10^{-2}	
chr10:59756803	cucucu	cucugg	2.027	2.13×10^{-2}	
chr10:59756810	cucucu	cucccu	2.58	4.94×10^{-3}	
chr10:59756817	cucucu	cucccu	2.652	4.00×10^{-3}	
chr10:59756837	cucucu	aucccu	2.098	1.80×10^{-2}	
chr10:59756841	cucucu	cugacu	2.107	1.76×10^{-2}	
chr10:59756850	cuuucu	auuucc	2.444	7.26×10^{-3}	
chr10:59756855	cucucu	cuguuu	1.911	2.80×10^{-2}	
chr10:59756858	cuuucu	uuuuau	2.185	1.44×10^{-2}	
chr10:59756860	cuuucu	uuaucu	2.383	8.59×10^{-3}	
chr10:59756860	cucucu	uuaucu	1.911	2.80×10^{-2}	
chr10:59756862	cuuucu	aucucu	2.333	9.82×10^{-3}	
chr10:59756862	cucucu	aucucu	2.277	1.14×10^{-2}	
chr10:59756745	acuaay	auuaau	2.793	2.61×10^{-3}	
chr10:59756748	acuaay	aaucac	1.728	4.20×10^{-2}	QKI
chr10:59756731	wgcaugm	agcaugu	2.592	4.77×10^{-3}	
chr10:59756764	wgcaugm	uccaugu	1.895	2.90×10^{-2}	RBFOX1
chr10:59756796	wgcaugm	ugcaugc	3.171	7.60×10^{-4}	
chr10:59756732	gcaug	gcaug	1.694	4.51×10^{-2}	
chr10:59756556	cuuucu	ucuucu	2.309	1.05×10^{-2}	RBFOX2

B

Human					
Genomic Coordinate	Motif	K-mer	Z-score	P-value	
chr10:72524892	cuuucu	auuucc	2.213	1.34×10^{-2}	PTB
chr10:72524866	ucuu	uauu	2.17	1.50×10^{-2}	
chr10:72524863	ucuu	uauu	2.161	1.53×10^{-2}	
chr10:72524854	ucuu	uuuu	2.17	1.50×10^{-2}	
chr10:72524847	ucuu	uguu	2.17	1.50×10^{-2}	
chr10:72524846	cuuucu	guuucu	2.2	1.39×10^{-2}	
chr10:72524843	ucuu	ucuu	2.723	3.23×10^{-3}	
chr10:72524842	cuuucu	cuuugu	2	2.27×10^{-2}	
chr10:72524835	ucuu	uauu	2.161	1.53×10^{-2}	
chr10:72524830	ucuu	uguu	2.161	1.53×10^{-2}	
chr10:72524828	cuuucu	uuuugu	2.213	1.34×10^{-2}	
chr10:72524828	ucuu	uuuu	2.143	1.61×10^{-2}	
chr10:72524776	ucuu	uguu	2.455	7.04×10^{-3}	
chr10:72524775	cuuucu	guuuuu	2.712	3.34×10^{-3}	
chr10:72524774	cuuucu	uuuuuu	2.812	2.46×10^{-3}	
chr10:72524774	ucuu	uuuu	2.455	7.04×10^{-3}	
chr10:72524773	ucuu	uuuu	2.455	7.04×10^{-3}	
chr10:72524772	cuuucu	uuuucu	3.45	2.80×10^{-4}	
chr10:72524772	ucuu	uuuu	2.455	7.04×10^{-3}	
chr10:72524769	ucuu	ucuu	3.286	5.08×10^{-4}	
chr10:72524768	cuuucu	cuuuuu	3.2	6.87×10^{-4}	
chr10:72524767	ucuu	uuuu	2.455	7.04×10^{-3}	
chr10:72524766	cuuucu	uuuucu	3.613	1.51×10^{-4}	
chr10:72524766	ucuu	uuuu	2.455	7.04×10^{-3}	
chr10:72524763	ucuu	ucuc	2.455	7.04×10^{-3}	
chr10:72524761	ucuu	ucau	2.455	7.04×10^{-3}	
chr10:72524758	cuuucu	ucuucu	3	1.35×10^{-3}	
chr10:72524758	ucuu	ucuu	3.286	5.08×10^{-4}	
chr10:72524755	ucuu	ucug	2.455	7.04×10^{-3}	
chr10:72524745	cuuucu	auuucu	3.175	7.49×10^{-4}	
chr10:72524742	ucuu	ucug	2.161	1.53×10^{-2}	
chr10:72524630	cucucu	cucccu	2.088	1.84×10^{-2}	
chr10:72524616	cuuucu	auuucg	2.038	2.08×10^{-2}	
chr10:72524607	cuuucu	cuguca	2.3	1.07×10^{-2}	
chr10:72524607	cucucu	cuguca	1.894	2.91×10^{-2}	
chr10:72524605	cucucu	gucacu	1.894	2.91×10^{-2}	
chr10:72524602	ucuu	acuu	2.143	1.61×10^{-2}	
chr10:72524853	acuaay	uuuaac	2.011	2.22×10^{-2}	QK1
chr10:72524732	acuaay	acugac	2.587	4.84×10^{-3}	
chr10:72524708	acuaay	aauuac	2.554	5.32×10^{-3}	
chr10:72524683	acuaay	aguaau	2.5	6.21×10^{-3}	
chr10:72524595	acuaay	auuaac	3.109	9.39×10^{-4}	
chr10:72524721	wgcaugm	agcaugu	3.053	1.13×10^{-3}	RBFOX1
chr10:72524656	wgcaugm	cgcauga	3.474	2.56×10^{-4}	
chr10:72524649	wgcaugm	ugcauau	2.487	6.44×10^{-3}	
chr10:72524720	gcaug	gcaug	1.694	4.51×10^{-2}	RBFOX2

Figure S3. Prediction of the binding sites of splicing factors involved in muscle differentiation in *Homo sapiens* and *Mus musculus* 300 bp surrounding the *MICU1.1* extra-exon. (**A**,**B**) Genomic coordinates, motives, K-mer, Z-score and P-value of the identified binding sites. (**A**) Binding sites in the mouse sequence. (**B**) Binding site in the human sequence.

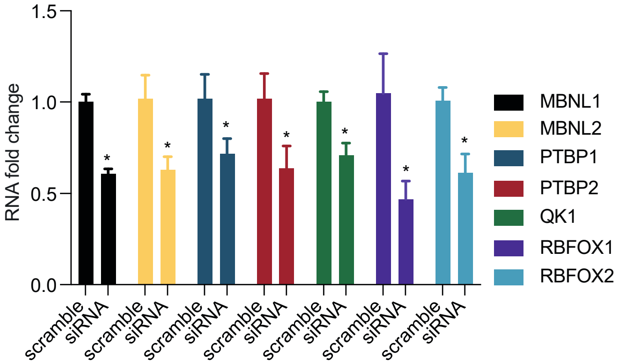

Figure S4. Silencing efficiency of splicing factors involved in myogenic differentiation. C2C12 myoblasts were transfected with either control scramble or the indicated siRNA. After 72 hours, cells were harvested, and expression levels were evaluated by qPCR and normalized to GAPDH. n=3. Data are expressed as mean ± SEM. For data analysis, parametric one–way ANOVA was used with post hoc Tukey's multiple comparison test for each sample. * $p \leq 0.05$.
